# Regulatory T Cell Modulation by *Lactobacillus rhamnosus* Improves Feather Damage in Chickens

**DOI:** 10.3389/fvets.2022.855261

**Published:** 2022-04-11

**Authors:** Claire Mindus, Nienke van Staaveren, Dietmar Fuchs, Johanna M. Gostner, Joergen B. Kjaer, Wolfgang Kunze, M. Firoz Mian, Anna K. Shoveller, Paul Forsythe, Alexandra Harlander-Matauschek

**Affiliations:** ^1^Department of Animal Biosciences, Ontario Agricultural College, University of Guelph, Guelph, ON, Canada; ^2^Biocenter, Institute of Biological Chemistry, Medical University of Innsbruck, Innsbruck, Austria; ^3^Biocenter, Institute of Medical Biochemistry, Medical University of Innsbruck, Innsbruck, Austria; ^4^Institute of Animal Welfare and Animal Husbandry, Friedrich-Loeffler-Institut, Celle, Germany; ^5^Brain-Body Institute, St. Joseph's Healthcare, McMaster University, Hamilton, ON, Canada; ^6^Division of Respirology, Department of Medicine, McMaster University, Hamilton, ON, Canada; ^7^Department of Medicine, Faculty of Medicine and Dentistry, University of Alberta, Edmonton, AB, Canada

**Keywords:** psychoneuroimmunology, microbiota, social interaction, kynurenine, aromatic amino acids, laying hen

## Abstract

It is currently unclear whether potential probiotics such as lactic acid bacteria could affect behavioral problems in birds. To this end, we assessed whether a supplementation of *Lactobacillus rhamnosus* JB-1 can reduce stress-induced severe feather pecking (SFP), feather damage and fearfulness in adult birds kept for egg laying. In parallel, we assessed SFP genotypic and phenotypic-related immune responses and aromatic amino acid status linked to neurotransmitter production. Social stress aggravated plumage damage, while *L. rhamnosus* treatment improved the birds' feather cover in non-stressed birds, but did not impact fearfulness. Our data demonstrate the significant impact of *L. rhamnosus* supplementation on the immune system. *L. rhamnosus* supplementation induced immunosuppressive regulatory T cells and cytotoxic T cells in both the cecal tonsils and the spleen. Birds exhibiting the SFP phenotype possessed lower levels of cecal tonsils regulatory T cells, splenic T helper cells and a lower TRP:(PHE+TYR). Together, these results suggest that bacteria may have beneficial effects on the avian immune response and may be useful therapeutic adjuncts to counteract SFP and plumage damage, thus increasing animal health and welfare.

## Introduction

Poultry is the most extensively farmed land animal totalling approximately 26 billion birds worldwide in 2019 alone (1). Severe feather pecking (SFP) is a behavior commonly observed in birds kept for egg-laying, where female hens forcefully peck, remove and sometimes eat feathers of conspecifics (2). While some pecking is part of their natural behavior, SFP is a major behavioral problem as it causes feather cover damage and can develop into cannibalism (2). Birds rely on intact feather cover for thermoregulation/insulation and water-proofing (3), locomotion, and navigation of the environment (4, 5), and social communication (6). SFP is deleterious to the health and welfare of farmed birds. In addition, damage to the feather cover can lead to significant economic losses for commercial farms, for example through increased feed consumption to compensate for energy losses due to reduced feather cover (2). Flock mortality resulting from skin injuries and cannibalism events are also a non-negligible source of financial loss and can negatively impact consumers' trust and acceptance of poultry farming (2). Current farming practices aim to reduce consequences of the behavior rather than the behavior itself (7). Such practices, like beak trimming, are under increasing scrutiny because of animal welfare, ethical and societal concerns. Consequently, some commonly used procedures are being banned in multiple countries (7).

Despite decades of research, the cause of SFP is still unknown, attesting to the multifactorial nature of this behavior (2). Indeed, SFP can be influenced by physical and social environmental factors (8), as well as genetics (9), stress coping mechanisms (10), fearfulness (11), and neurobiology as determined by the monoaminergic systems (12, 13) or the immune system (14, 15). Of the multiple comorbidities associated with SFP, the involvement of the gut in the development of the behavior has gained attention. Descendants of White Leghorn pedigree lines that are bred for high or low SFP activity (9) are consistently reported to host distinct gut microbiota and short-chain fatty acid profiles (16–19). For instance, Birkl et al. (18) and van der Eijk et al. (19) found a lower abundance of *Lactobacillus* species in the cecal excreta of birds genetically selected for SFP behavior.

Lactobacilli are the predominant bacterial genus throughout the gastro-intestinal tract of chickens (20–23). Evidence suggests that they influence the gut-brain axis communication via an immune-mediated humoral pathway and a neural route (24–27). Lactobacilli are thought to impart beneficial effects on the stress response, the immune system, and stress-induced behavior in a diverse set of species (26, 28–31). For example, Lactobacillus supplementation increases T lymphocyte subpopulations in the gastro-intestinal tract of chicks, thereby impacting inflammatory processes (32). They also modulate the catabolic pathways of the aromatic amino acids (AAA), tryptophan (TRP) (33–35), phenylalanine (PHE), and tyrosine (TYR) (36). These AAAs are the precursors of kynurenine (KYN) and the monoaminergic neurotransmitters serotonin and dopamine, respectively (37, 38). The activities of the enzymes responsible for the TRP to KYN and PHE to TYR conversions are approximated by plasma KYN:TRP and PHE:TYR, respectively (39, 40). The TRP: (PHE+TYR) is a surrogate parameter reflecting the competition of TRP with other AAAs for uptake across the blood-brain-barrier (38).

Interestingly, the aforementioned physiological pathways influenced by lactobacilli are also interlinked with SFP. When considered together, these data suggest a gap in our understanding about the effects of the gut microbiome on SFP. We conducted a first study in adult hens selected for high SFP activity and showed that continuous oral intake of Lactobacillus rhamnosus improved feather cover, prevented stress-induced SFP behavior, changed regulatory T cell populations, and limited cecal microbiota dysbiosis (41). In a second study, the same L. rhamnosus strain was administered to chicks/pullets housed in large groups of low and high SFP genetic lines. Birds received the supplementation during early life to determine its efficacy as a preventative measure for SFP development under chronic stress. We found that L. rhamnosus caused a short-term increase in plasma TRP and TRP:(PHE+TYR), as well as an increase in all T lymphocytes of the spleen and cecal tonsils (42). L. rhamnosus is reported to modify gut motility within minutes of exposure ex vivo in mice (43) and chickens (44) and can reverse acute restraint stress-induced intestinal motility in mice (45). This demonstrates that Lactobacillus signaling can occur independently of colonization, alteration of the microbiome composition, or other longer-term adjustments (43). As such, it might be used as an immediate treatment against stress.

Consequently, the present study aimed to (1) evaluate the immediate impact of the oral administration of *L. rhamnosus* in response to stress by monitoring feather condition, fear behavior, and the immune and monoaminergic precursor responses in large mixed groups of low and high SFP laying hens, and (2) determine whether these physiological parameters are interrelated with the genetic background and the SFP phenotype of birds. To this end, we measured feather damage, SFP behavior, fear behavior, immunological markers, and actors (T-cells profiles, KYN:TRP ratio, and nitrite level) and markers of AAA metabolism (TRP, PHE, TYR, and their respective ratios).

## Materials and Methods

### Ethical Statement

The experiment was approved by the University of Guelph Animal Care Committee (Animal Utilization Protocol #4113). To promote both refinement and reduction of bird numbers, the lines shared an experimental unit/pen (46).

### Animals and Housing

Three pedigree lines of White Leghorn laying hens are maintained since 2015 at the University of Guelph Research Station (Guelph, Ontario, Canada). Yearly, this breeding flock is divergently selected for high (HFP) and low (LFP) severe feather pecking (SFP) activity or kept as unselected controls (UC) (9). Eggs were incubated, hatched in separate compartments per pedigree mother hen. At hatch, a total of 311 non-beak trimmed chicks were individually wing-tagged, and systemically allocated to 12 identical pens of 25 ± 2 birds each (8 ± 1 birds of each line; mixed lines per pen) in a windowless room. Each floor pen (1.6m^2^) was littered with wood shavings and contained one round metal feeder (43 Ø cm), a drinker line (7 nipples), an A-frame perch (15 cm of perch/hen, 55 and 120 cm above the ground), and three nest boxes. The birds were able to hear other birds in neighboring pens, but visual contact was prevented by opaque PVC boards between the pens. Light was provided at 20 Lux from 05.00 h till 19.00 h and average daily temperature was 20 °C. Birds had *ad libitum* access to water and corn/wheat/soybean meal based feed (University of Guelph Research Station starter [0–6 weeks], grower [7–16 weeks] and layer [>17 weeks] mash diet) and housed under conventional management conditions at the research station. At 15 weeks of age (woa), one bird had to be put down due to cannibalism by conspecifics.

### *Lactobacillus rhamnosus* Supplementation and Stress Treatment

**Figure 1:**
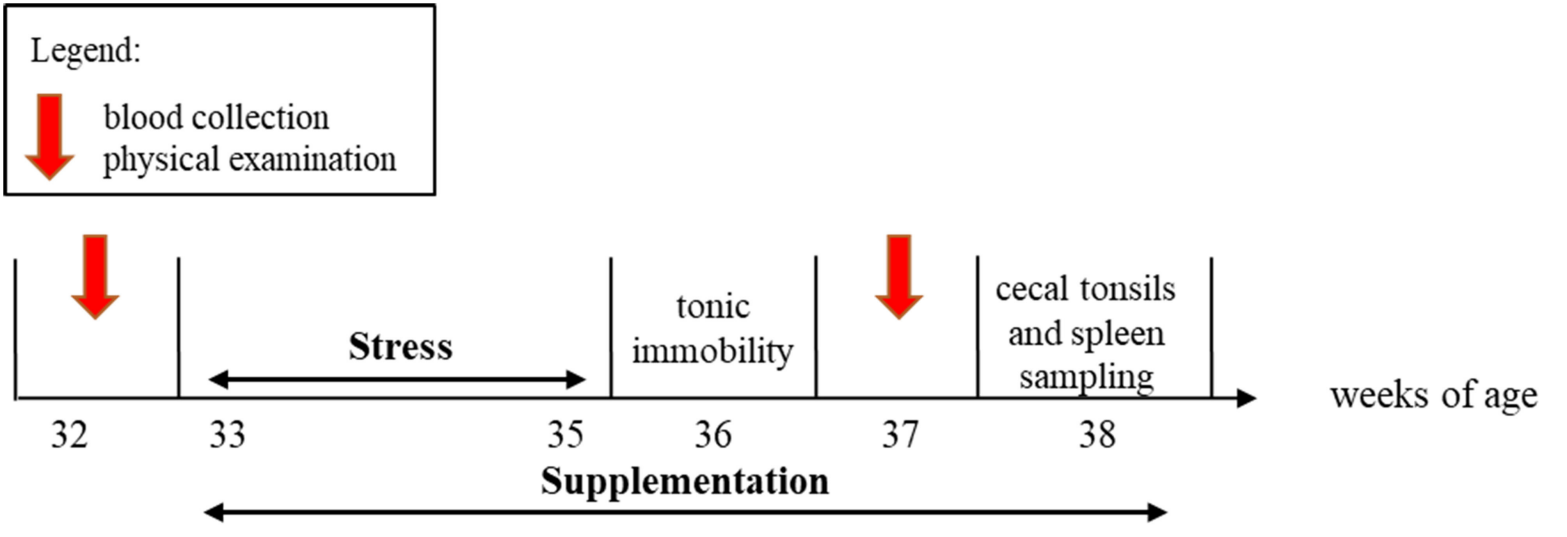
Scheme of the experimental timeline. The *L. rhamnosus* or Placebo supplementation started at 33 weeks and lasted 6 weeks. The stress treatment spanned weeks 33–35. Physical examinations and blood collection were conducted at 32 and 37 weeks of age (woa). Tonic immobility was conducted at 36 woa. Spleen and cecal tonsils samplings were performed at 38 woa.

An overview of the experimental timeline is presented in Figure 1. From 33 to 38 woa, six pens were systematically assigned to receive an oral supplementation with either *L. rhamnosus* JB-1™ (Lacto, n = 6 pens, 157 birds) dissolved in drinking water or a placebo of drinking water (Placebo, n = 6 pens, 154 birds). *L. rhamnosus* JB-1™ was a gift from Alimentary Health Inc., Cork, Ireland to Paul Forsythe and Wolfgang Kunze, McMaster University.

Employing a supplementation method that can be easily adopted in a farm setting, birds were supplemented as a group within each pen. Supplementation was provided daily (Monday to Friday) between 9:00 h to 10:30 h. The Lacto treatment was prepared by dissolving 5 x 10^9^ Colony Forming Units (CFU) of *L. rhamnosus* JB-1™ into 19 mL of warm drinking water per bird. To encourage birds to drink during the supplementation period, the drinker lines were raised to prevent water access for 1 h prior to supplementation. The Lacto or Placebo treatment were provided to the birds in their home pen using two round plastic 1L-drinkers (averaging 475 ± 38 mL per pen). Drinkers were monitored until they were voluntarily emptied (~10 min), after which they were removed from the pens. Subsequently, the original drinker lines were lowered until the next round of supplementation.

At 33 woa, concomitant with the beginning of the supplementation treatment, three pens of each supplementation type were systematically assigned to a stress regimen in an attempt to induce SFP (13). The stress regimen lasted for 3 weeks (stress, *n* = 156 birds). The remaining three pens of Lacto and Placebo birds were left undisturbed (non-stress, *n* = 155 birds). Stressors were environmental (removal of perches and shavings, as well as blocking nest-boxes from Monday to Friday), and social (social disruption by mixing). Social disruption was repeated 3–4 times per week in the afternoon (14:00–17:00 h) for a total of 10 events. Stressed pens were split into two subgroups of three to four individuals and mixed with another subgroup from a different pen in the stress treatment assigned to the same supplement type (Lacto or Placebo). Upon mixing, birds were placed in a new, but identical pen to create a new environment for all birds. Wood shavings from the stressed pens were removed during the first mixing. This stress regimen was designed to mimic the unpredictable and repeated stressors that hens encounter in commercial farm settings (13, 47).

### Behavioral Observations and Feather Damage Scoring

Prior to the experiment, birds were individually identified using continuously numbered silicone backpacks (8 x 6 x 0.5 cm) fastened onto the hens around the wings via two elastic straps secured to the backpacks with metal eyelets (48). Behavioral observations were conducted in the home pens via video recordings scheduled outside of the working hours of the farm staff to avoid any human bias. Cameras (Samsung SNO-5080R, IR, Samsung Techwin CO., Gyeongi-do Korea) were ceiling-mounted ahead of the trial to obtain a full view of each pen.

After determining the time windows during pilot observations, each pen was video recorded 10 min in the morning for a total of 100 min between 32 and 38 woa: 2 days at 32 woa as baseline, three days during the supplementation and stress treatments (35 woa), and 5 days post-stress treatment (36-38 woa). Behavioral recordings totaled 16 h of video and analysis was done by five trained blinded observers (Pearson's correlation of 0.88 for intra-observer reliability and 0.75 for inter-observer reliability) (42). All-occurrence sampling was used on all 311 birds to record the actor and recipient of SFP. SFP was defined as intent forceful peck(s) toward the feathers/body of conspecifics that may remove feathers or cause injury (2). All birds were individually examined for feather cover damage and bodyweight in weeks 32 and 37 by four blinded operators. Plumage damage to the neck, back and tail was assessed using a scoring scale adapted from Decina et al. with reported Kappa coefficients of 0.6-0.9 (49). The severity of plumage damage ranked from 0 (no or slight wear, nearly intact feathering) to 2 (at least one featherless area ≥ $2 Canadian coin, diameter: 28 mm). A fourth score (3) was added to account for the presence of at least one featherless area ≥ $2 Canadian coin with fresh blood stains.

### Tonic Immobility

A tonic immobility test was performed by four blinded operators over 3 days during week 36 as described by Jones and Faure (50) as a measure of fearfulness. Birds were individually removed from their home pen and tested in a nearby separate room. Birds were placed on their back in a U-shaped plastic cradle covered with a dark fabric. A standing operator then induced tonic immobility by gently restraining the bird for 15 s with one hand over the bird's breast and the other over the head. The induction was considered successful if the bird remained motionless for at least 10 s. After a successful induction, the operator sat and recorded the duration of tonic immobility, i.e., latency to self-righting. Birds were induced a maximum of three times, and the number of inductions needed was recorded. Birds still in tonic immobility after 5 min were given the maximum duration of 5 min.

### Blood Sampling and Amino Acid Analysis

Blood samples were collected at 32 and 37 woa, from the wing vein using EDTA-coated vacutainer tubes (2 mL/hen). Individual birds were sampled within 1 h after their last meal on the same day of the week and at the same time of day (between 10:00 and 14:00 h) for both sampling points. Samples were gently inverted and stored on ice immediately after collection (maximum of 4 h). Plasma was separated by centrifugation at 4°C, 1,780 *g* for 15 min and stored at −80°C until further analysis.

The concentration of aromatic amino acids tryptophan (TRP), its derivative (kynurenine [KYN]), phenylalanine (PHE) and tyrosine (TYR), and nitrite were determined as reported previously (51). In brief, samples were analyzed via reversed-phase HPLC. The TRP, PHE and TYR concentrations were determined by monitoring their natural fluorescence (TRP: excitation [Ex] wavelength [λ] 286 nm, emission [Em] [λ] 366 nm; PHE, TYR: Ex λ 210 nm, Em λ 302 nm).

In mammals, the KYN to TRP ratio can be used to estimate TRP metabolism along the KYN axis, along which 90% of TRP not used for protein synthesis is catabolized. In humans, this ratio is used as an index of the IDO-1 activity if accompanied by an increase of immune activation markers such as neopterin (39). The PHE to TYR ratio may be used as a surrogate of phenylalanine 4-hydroxylase (PAH) activity, which converts PHE to TYR (40). TRP:(PHE+TYR) is a substitution for the commonly used ratio of TRP to large neutral amino acids. As described in Wurtman et al. (38), this ratio represents the competition of TRP with other amino acids for uptake across the blood-brain-barrier. However, it should be acknowledged that poultry physiology differs from mammals because of potential evolutionary variations (52, 53) and thus, the results should be approached with caution. As a surrogate marker of nitric oxide (NO) production, the stable NO metabolite nitrite was measured using a modified Griess assay (Merck KGaA, Darmstadt, Germany) (51).

### Immune Phenotype

At week 38, 60 hens (five hens per line x supplementation type x stress treatment groups) were put down by cervical dislocation to determine T-cell populations as described in Mindus et al. (41). In brief, one cecal tonsil and the spleen were harvested from each bird within 3 min after death and kept in 5 mL of 5% fetal bovine serum (FBS) containing RPMI medium. Cells from both tissues were isolated, suspended, centrifuged, and counted. Viable spleen and cecal tonsils cells were diluted in fluorescence-activated cell sorting (FACS) buffer (PBS + 2% FBS) to a concentration of 10^6^ cells/ml. Both splenocytes and cecal tonsil cells were stained for T-helper cells (CD3^+^CD4^+^ T cells), cytotoxic T lymphocytes (CD3^+^CD8^+^ T cells), and regulatory T cells (CD4^+^CD25^+^ T cells) markers using the same antibodies as in Mindus et al. (41). Data were acquired using FACSCelesta (Becton Dickinson, Oakville, ON, Canada) and analyzed by FlowJo (BD Bioscience, Ashland, OR, USA).

### Statistical Analysis

FP frequencies were determined per individual per 10 min. Due to the low frequency of SFP, we focused on feather damage as a reliable indicator of the intensity of the behavior (54). The neck, back, and tail feather cover scores were used to assign a general plumage damage score (0–3; maximum score of the three body areas) for each bird at each sampling point. However, to further identify the physiological pathways linked to the behavior, we categorized birds as SFP peckers based on whether or not they had performed the behavior throughout the course of the experiment regardless of their genotype. Birds exhibiting at least one severe feather peck at 32, 35 or between 36–38 woa were categorized as severe peckers. Birds that performed 0 pecks were categorized as non-severe peckers.

The SAS software (version 9.4, SAS Institute, Cary NC) was used for all statistical computations. Unless specified, generalized linear mixed models (PROC GLIMMIX) were used to analyze the data. The assumptions of normally distributed residuals and homogeneity of variance were examined graphically with the use of QQ plots. Scatter plots of studentized residuals against predicted values and treatment values, and a Shapiro-Wilk test of normality were used to confirm the assumptions of the variance analysis. To detect possible outliers, studentized residuals outside a ± 3.4 envelope were used. Data was transformed where necessary. Least square (LS) means and standard errors on the data scale were recovered using the ilink option. Values are presented as LS means ± standard error, unless stated otherwise. Differences between means were compared pairwise using a Tukey-Kramer adjustment. Statistical significance was considered at *P* < 0.05.

Variances of plumage damage, bodyweight, tonic immobility duration and number of inductions to trigger tonic immobility, each T cell subset proportion, aromatic amino acid (AAA), KYN and ratios were partitioned into the fixed effect of supplementation, stress, line and their interaction with the best fitted distribution and their significance were tested through F-tests. When possible, the baseline values (collected at 32 woa) were used as covariates and the pens of the birds were designated as a random effect except for the tonic immobility outcomes (whereby observer within a day, the day and the pen were designated as random effects).

Finally, additional models were performed to identify whether physiological measurements were interrelated with the SFP phenotype. Variance of each T cell subset (obtained at 38 woa) and each AAA, their metabolites and ratios (obtained at 37 woa) was partitioned with the SFP phenotype (characterized from the behavior displayed from week 35 until week 37 [AAA variables determined from blood sampling] or week 38 [T cell subsets collected from tonsil/spleen sampling]) as a fixed effect and pen as a random effect.

## Results

### Stress Aggravates Plumage Damage While *L. rhamnosus* Supplementation Improves Feather Cover Under Non-stressful Conditions

We assessed if the oral treatment with *L. rhamnosus* (Lacto) reduced stress-induced damage to the feather cover. We report that the stress treatment alone aggravated the severity of the overall feather damage (F_1,294_ = 3.98, *P* < 0.05, Table 1). Indeed, 74% of stressed birds had clear evidence of feather loss (score >2) compared to only 50% of non-stressed birds. Overall, the Lacto treatment favored the odds of less feather damage as only 51% of Lacto had clear evidence of feather loss (score >2) relative to 74% in the Placebo group (F_1,294_ = 3.22, P = 0.074, Table 1). However, there was a significant interaction between Lacto and stress treatments on the severity of plumage damage (F_1,294_ = 27.30, *P* < 0.001). Modeling the probability of birds having a lower score (i.e., more intact feather cover), we found that the Lacto treatment did significantly reduce plumage loss under non-stressed conditions. Indeed, the Lacto non-stressed birds were more likely to show more intact feather cover compared to the Placebo non-stressed (OR = 5.24, 95%CI = 2.62–10.46) and the Lacto-stressed birds (OR = 5.50, 95%CI = 2.72–11.08). This was reversed in the Placebo group, as Placebo stressed birds were more likely to present more intact feather cover than the Placebo non-stressed birds (OR = 2.1, 95%CI = 1.17–3.94). Finally, Lacto treatment did not improve feather cover under stressful condition as Lacto stressed birds were less likely to show intact feather cover (OR = 0.44, 95%CI = 0.24 - 0.82) than the Placebo stressed birds. With respect to the genetic lines, birds from the HFP line tended to be more likely to have more intact feather cover than the birds from the LFP line (Means ± SD, HFP:1.5 ± 1.08 vs. LFP 2.0 ± 0.99; OR = 1.74, 95%CI = 1.01–2.99).

**Table 1:** Percentage of birds receiving a given score for overall plumage damage within each treatment group.

**Treatment**	**Class**	**Percentage of birds for each score (%)**				**OR**	**95% CI**	***P*-value**
		**0**	**1**	**2**	**3**			
Supplementation	Placebo	10	16	42	32	Ref	Ref	0.074
	Lacto	33	16	21	29	1.53	0.96–2.42	
Stress	NS	32	18	23	27	Ref	Ref	0.047
	S	11	15	39	35	0.63	0.39–0.99	
Supplementation x Stress	S-Lacto	13	10	30	47			<0.001
	S-Placebo	9	19	48	23			
	NS-Lacto	55	22	12	12			
	NS-Placebo	10	13	35	42			

*The birds' plumage condition was assessed on the neck, tail, and back area (scale of 0 to 3; higher score indicating more severe damage) at 37 weeks of age. The maximum score from these areas was retained as the overall plumage condition. The odds ratio (OR) and 95% Confidence Interval (CI) were modeling the probability of a lower overall score (i.e., more intact feather cover). Birds received a supplementation treatment (water [Placebo] or L. rhamnosus [Lacto] supplementation, weeks 33–38) and stress treatment (non-stressed [NS] or stressed [S], weeks 33–35). Number of birds: S-Lacto = 79, S-Placebo = 77, NS-Lacto = 78, NS-Placebo = 77. Ref = Reference value*.

### *L. rhamnosus* Treatment Can Buffer Against Bodyweight Loss but Does Not Affect Fearfulness

We determined the effects of Lacto treatment and stress on bodyweight and its implications for fear behavior as measured through the duration of immobility and number of inductions in a tonic immobility test. We found that Lacto treatment prevented stress-induced bodyweight loss. Indeed, there was a significant interaction between Lacto and stress treatment in determining bodyweight (F_1,296_ = 6.11, *P* = 0.014). Placebo non-stressed birds were 3% heavier compared to the Placebo stressed birds (Placebo non-stressed: 1.77 ± 0.008 vs. Placebo stressed 1.72 ± 0.008 kg, *P* < 0.001). We observed no significant difference in bodyweight between stressed and non-stressed birds in the Lacto birds (Lacto non-stressed: 1.75 ± 0.008 vs. Lacto stressed 1.74 ± 0.008 kg, *P* > 0.05).

The number of inductions required to enter tonic immobility and its duration were not impacted by the Lacto treatment, stress or by their interaction (*P* > 0.05, Supplementary Table 1). However, HFP birds had significantly shorter tonic immobility durations (HFP: 60 ± 9.5 s vs. LFP: 89 ± 13.8 s, F_2, 278_ = 3.69, *P* = 0.026) and necessitated more inductions for tonic immobility (HFP: 1.7 ± 1.01 vs. LFP: 1.4 ± 0.77, F_2,283_ = 0.03, *P* = 0.006) than LFP birds, while UC birds showed a more intermediate response (duration: 72 ± 11.2 s, inductions: 1.6 ± 0.89).

### Oral Treatment of *L. rhamnosus* Induces a Strong Regulatory T Cell Response

**Figure 2:**
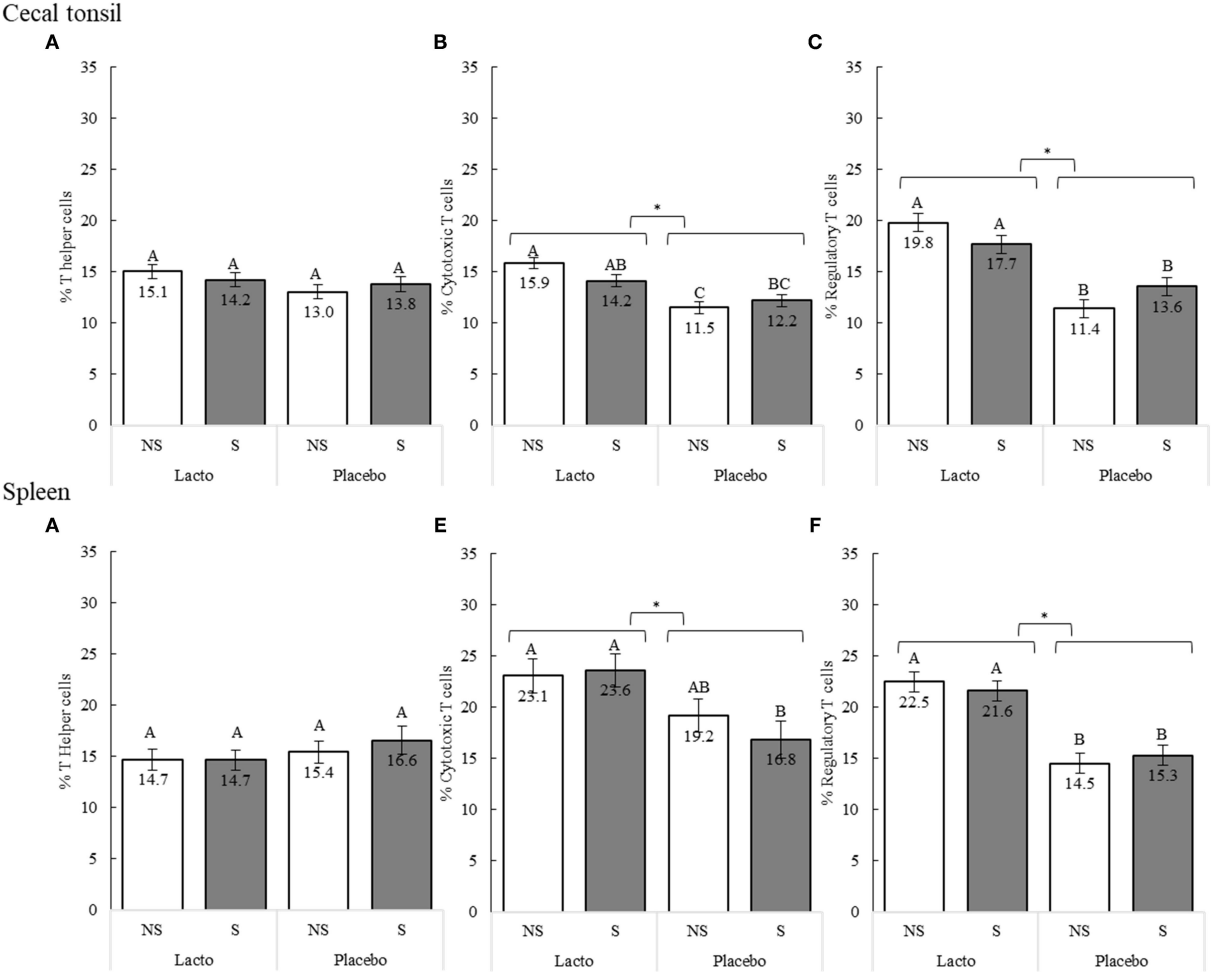
Proportions of T cell sub-populations in the cecal tonsils **(A–C)** and spleen **(D–F)** of 38-weeks old birds after 5 weeks of supplementation (*L. rhamnosus* [Lacto] or water [Placebo] supplementation, 33–38 weeks of age) and 3 weeks of stress treatment (non-stressed [NS] or stressed [S], 33–35 weeks of age). Sub-populations were identified using the following combinations of cell surface markers: T helper cells = CD3^+^CD4^+^; cytotoxic T cells = CD3^+^CD8^+^; T regulatory cells = CD4^+^CD25^+^ (n of birds: S-Placebo = 15, NS-Placebo = 15, S-Lacto = 15, NS-Lacto = 15). Different superscript letters indicate statistically significant differences between specific supplement and stress treatment comparisons of the interaction, and ^*^ indicates statistically significant differences of the supplementation treatment as a main effect (*P* < 0.05).

In poultry species, chronic stress decreases the proportion of peripheral blood lymphocytes (55), an outcome that can be countered by lactobacilli (32, 41, 42). We assessed the capacity of L. rhamnosus treatment to stimulate T helper cells (CD3^+^CD4^+^ T lymphocytes), cytotoxic T cells (CD3^+^CD8^+^ T lymphocytes), and regulatory T (Treg) cells (CD3^+^CD4^+^CD25^+^ T lymphocytes) in the spleen and cecal tonsils of laying hens in response to a stress treatment induced between 33 and 35 woa (Figure 2).

The Lacto and stress treatments interacted to determine the proportion of the cytotoxic T cells (F_1,47_ = 4.30, P = 0.044, Figure 2B) and Treg cells (F_1,47_ = 5.85, P = 0.020, Figure 2C) in the cecal tonsils. While the Lacto supplementation generally increased the proportions of these cells compared to the Placebo, the difference was larger in non-stressed groups. In contrast, a significant increase of splenic cytotoxic T cells (Figure 2E) was only observed in the stressed groups (P = 0.009), while no significant difference was found between the non-stressed groups (P = 0.222). The stress treatment alone did not influence T cell proportions (*P* > 0.05). However, overall, Lacto treatment increased the proportion of Treg cells in the cecal tonsils (F_1,47_ = 51.40, *P* < 0.001) and spleen (F_1,47_ = 53.91, *P* < 0.001), as well as the proportion of cytotoxic T cells in both tissues (cecal tonsil, F_1,47_ = 29.26, *P* < 0.001; spleen, F_1,47_ = 13.94, *P* < 0.001) compared to Placebo birds (Figure 2). The proportion of T helper cells was not affected by the Lacto and stress treatment, or their interaction (*P* > 0.05).

To better understand the physiological pathways underlying SFP behavior, the interrelatedness of the immune response with the genetic lines and the SFP phenotype from week 35 to week 38 (tissue collection) were evaluated. We found that lines reacted differently to the Lacto and stress treatments (Table 2). Indeed, Lacto treatment increased the proportion of Treg cells in the HFP and UC birds compared to the Placebo in the tonsils (F_2,47_ = 6.12, *P* < 0.01), and to a lesser extent the spleen (F_2,47_ = 12.89, *P* < 0.01). No difference in Treg cells was observed in the LFP birds following Lacto treatment (Table 2). Inversely, Lacto birds had a higher proportion of splenic cytotoxic T cells in LFP birds compared Placebo birds (P = 0.049), while no difference was observed in the other lines (P>0.05, Table 2). Stress increased the proportion of splenic Treg cells in the LFP line compared to non-stressed birds (*P* = 0.035). No difference was observed in the other lines (*P* > 0.05, Table 2). Stressed HFP birds had fewer tonsil Treg cells than stressed LFP birds (*P* = 0.027), while no difference was observed in the non-stress birds (*P* = 0.924, Table 2).

**Table 2:** Least squares means (± standard error) of the proportions of T cell sub-populations in the spleen and cecal tonsils in 38 weeks old birds according to their genetic line (UC: unselected control, LFP: low feather pecking line, HFP: high feather pecking line).

	**Lacto**			**Placebo**			
	**UC**	**LFP**	**HFP**	**UC**	**LFP**	**HFP**	**Supplementation x line**
	**(*n* = 10)**	**(*n* = 10)**	**(*n* = 10)**	**(*n* = 10)**	**(*n* = 10)**	**(*n* = 10)**	**F- Statistic, *P*-value**
**Cecal tonsils**							
T helper cell	15.2 ± 0.82	13.9 ± 0.83	14.9 ± 0.83	12.7 ± 0.83	14.4 ± 0.85	13.2 ± 0.85	F_2,47_ = 2.00, P = 0.147
Cytotoxic T cell	16.2 ± 0.70 ^a^	13.5 ± 0.70 ^ab^	15.2 ± 0.70 ^a^	11.1 ± 0.70 ^b^	13.4 ± 0.69 ^ab^	11.1 ± 0.69 ^b^	**F**_**2, 47**_ **=** **6.75, P** **=** **0.003**
Regulatory T cell	21.4 ± 1.08 ^a^	17.5 ± 1.08 ^ab^	17.5 ± 1.08 ^ab^	11.2 ± 1.08 ^c^	14.8 ± 1.08 ^bc^	11.5 ± 1.08 ^c^	**F**_**2, 47**_ **=** **6.12, P** **=** **0.004**
**Spleen**							
T helper cell	2.7 ± 0.08	2.7 ± 0.08	2.6 ± 0.08	2.8 ± 0.08	2.8 ± 0.09	2.7 ± 0.09	F_2,47_ = 0.29, P = 0.751
Cytotoxic T cell	23.4 ± 1.9 ^ab^	25.0 ± 1.9 ^a^	21.6 ± 1.9 ^ab^	18.1 ± 1.9 ^ab^	17.7 ± 2.01 ^b^	18.2 ± 2.03 ^ab^	F_2,47_ = 0.65, P = 0.526
Regulatory T cell	27.5 ± 1.19 ^a^	18.6 ± 1.19 ^b^	20.1 ± 1.19 ^b^	13.5 ± 1.19 ^c^	16.1 ± 1.19 ^bc^	15.1 ± 1.19 ^bc^	**F**_**2, 47**_ **=** **12.89**, ***P** **<*** **0.001**
							
	**Stress**			**Non-stress**			
	**UC**	**LFP**	**HFP**	**UC**	**LFP**	**HFP**	**Stress x line**
	**(*****n*** **=** **10)**	**(*****n*** **=** **10)**	**(*****n*** **=** **10)**	**(*****n*** **=** **10)**	**(*****n*** **=** **10)**	**(*****n*** **=** **10)**	**F- Statistic**, ***P*****-value**
**Cecal tonsils**							
T helper cell	12.8 ±0.82	14.9 ± 0.85	14.4 ± 0.85	15.1 ± 0.83	13.4 ± 0.83	13.6 ± 0.83	F_2,47_ = 3.05, P = 0.057
Cytotoxic T cell	12.8 ± 0.70	14.8 ± 0.69	11.9 ± 0.68	14.5 ± 0.69	12.1 ± 0.69	14.3 ± 0.69	**F**_**2, 47**_ **=** **7.39**, ***P** **<*** **0.001**
Regulatory T cell	15.2 ± 1.08 ^ab^	18.3 ± 1.08 ^a^	13.4 ± 1.08 ^b^	17.4 ± 1.08 ^ab^	14.0 ± 1.08 ^ab^	15.5 ± 1.08 ^ab^	**F**_**2, 47**_ **=** **5.84, P** **=** **0.005**
**Spleen**							
T helper cell	2.8 ± 0.08	2.8 ± 0.09	2.7 ± 0.09	2.7 ± 0.08	2.8 ± 0.08	2.6 ± 0.08	F_2,47_ = 0.21, P = 0.812
Cytotoxic T cell	19.1 ± 1.90	22.9 ± 2.01	18.6 ± 2.02	22.5 ± 1.93	19.7 ± 1.93	21.2 ± 1.91	F_2,47_ = 2.16, P = 0.127
Regulatory T cell	18.9 ± 1.19 ^abc^	20.0 ± 1.19 ^ab^	16.4 ± 1.19 ^bc^	22.0 ± 1.19 ^a^	14.7 ± 1.19 ^c^	18.8 ± 1.19 ^abc^	**F**_**2, 47**_ **=** **7.50, P** **=** **0.002**

*Birds underwent 5 weeks of supplementation (L. rhamnosus [Lacto] or water [Placebo] supplementation, 33–38 weeks of age) and 3 weeks of stress treatment (33–35 weeks of age). Sub-populations were identified using the following combinations of cell surface markers: T helper cells = CD3^+^CD4^+^; cytotoxic T cells = CD3^+^CD8^+^; T regulatory cells = CD4^+^CD25^+^.*

*Different superscript letters indicate statistically significant different comparisons within the interaction in each row at P <0.05.*

*F-Statistics and P-values of the supplementation x line or stress x line interaction; interaction terms significant at P <0.05 are bolded*.

We found that the genetic line alone impacts the proportion of splenic Treg cells (F_2,47_ = 4.24, P = 0.020, Supplementary Table 2). The UC line had a significantly higher proportion of Treg cells than the HFP (*P* < 0.05) and LFP (P = 0.032) lines; but there were no differences between the HFP and LFP birds (P = 0.981). Other proportions of T cells were similar between the genetic lines (*P* > 0.05, Supplementary Table 2). Phenotypical severe feather peckers (i.e., birds who performed at least one severe feather peck between 35-38 woa) were retrospectively identified and mostly came from the HFP (52%), UC (38%), and lastly LFP (10%) line as expected. Phenotypical severe feather peckers had reduced levels of Treg cells in the tonsils (F_1,53_ = 4.06, *P* = 0.049) and splenic T helper cells (F_1,53_ = 4.07, *P* = 0.049) compared to non-peckers (Supplementary Table 2).

### SFP Phenotype Is Associated With Lower TRP:(PHE+TYR) and Elevated TYR Levels

We investigated the impact of Lacto and stress treatments on the concentrations of aromatic amino acids (AAA), the TRP metabolite KYN, ratios, and nitrite at 37 weeks of age. *L. rhamnosus* supplementation, stressors, and their interaction did not significantly change peripheral plasma levels of TRP, PHE, TYR, KYN, and their relevant ratios. Furthermore, the TRP:(PHE+TYR) and nitrite concentrations were similar between groups (Supplementary Table 3).

We examined whether the AAA response was related with the genetic lines and SFP phenotype displayed from week 35 until week 37 (blood collection). Genetic line x stress interactions showed that LFP birds differ in their stress responses compared to birds from other lines (Supplementary Table 4). Indeed, in LFP birds, stress increased KYN concentrations (stressed: 0.38 ± 0.022 μM vs. non-stressed: 0.29 ± 0.021 μM, F_2,277_ = 4.69, *P* < 0.01) and KYN:TRP (stressed: 4.3 ± 0.25 μmol/mmol vs. non-stressed: 3.3 ± 0.25 μmol/mmol, F_2,277_ = 4.68, P = 0.01) levels while no change was observed in the stressed vs. non-stressed birds of the HFP and UC lines.

Overall, peripheral plasma levels of TRP, PHE, TYR, KYN, their relevant ratios, and the nitrite concentrations were similar between genetic lines (*P* > 0.05, Supplementary Table 5). Nevertheless, we report that phenotypic severe feather peckers had significantly lower TRP:(PHE+TYR) than birds that were not severe feather peckers (severe feather peckers: 0.35 ± 0.010 μmol/μmol vs. not severe feather peckers: 0.38 ± 0.007 μmol/μmol, F_1,292_ = 9.70, P = 0.002). Peckers also tended to have lower peripheral plasma TYR concentrations (severe feather peckers: 128 ± 3.5 μM vs. not severe feather peckers: 122 ± 2.2 μM, F_1,288_ = 3.72, *P* = 0.055).

## Discussion

The aim of the present study was to determine whether oral supplementation with a single *L. rhamnosus* strain can act as an immediate measure to reduce stress-induced severe feather pecking (SFP) and associated physiological changes. To this end, we assessed the feather cover, tonic immobility duration and number of inductions, T cells profiles, aromatic amino acids metabolism, along the kynurenine (KYN) and dopaminergic pathways quantified by plasma tryptophan (TRP), phenylalanine (PHE) and tyrosine (TYR) concentrations in laying hens following 5 weeks of supplementation (33–38 weeks of age [woa]) and a concomitant three-week stress regimen (33–35 woa). Three genetic lines of birds housed in mixed groups were used in this study: high feather pecking (HFP), low feather pecking (LFP) and unselected control (UC). We also analyzed the link between genotypic and phenotypic SFP behavior and physiological parameters. We report that stress aggravates the severity of damage to the feather cover while *L. rhamnosus* supplementation mitigated the feather damage in non-stressed conditions. Surprisingly, *L. rhamnosus* supplementation did not mitigate the damage to feather cover in stressed conditions. Furthermore, the *L. rhamnosus* supplementation induced immunosuppressive regulatory T cells (Treg) and cytotoxic T cells in both the cecal tonsils and the spleen. Birds exhibiting the SFP phenotype displayed lower levels of tonsil Treg and splenic T helper cells as well as a lower TRP:(PHE+TYR).

Considering the biological importance of feather cover (2), it is important to note that stress alone deteriorated the overall feather cover. Nearly 75% of stressed birds had clear evidence of feather loss, reflecting previous findings (13, 41). Most importantly, we found that the Lacto supplement tended to decrease the severity of the feather damage (Table 1), however, this effect was only found in non-stressed birds. Taken together, these results agree with mammalian studies in which *Lactobacillus* bacteria are known to have a positive influence in healthy, non-stressed individuals (29, 56, 57). Importantly, this finding was not replicated in the stressed birds where Lacto stressed birds actually had more feather damage than Lacto non-stressed birds (Table 1). Potentially, the current stress regimen overrode the potential beneficial effects of the supplementation in this study. Previous research using a more varied stress regimen (e.g., social disruption, shavings replacement, individual and group restraint, blocking nest boxes and perches in random order) showed that ingestion of *L. rhamnosus* positively modulates chronic stress-induced feather damage when continuously administered before, during and after stress to adult HFP birds (41). The fact that different results were observed in the current study when Lacto was supplemented only during and after stress, could highlight the importance of relative timing of probiotic supplementation. Interestingly, a recent meta-analysis showed that the order in which the *Lactobacillus*-based probiotics and stress treatments are applied does not change the effect of the probiotic in rodents (31). However, Liu et al. (58), showed that *L. rhamnosus* supplementation administered only post-social stress increased the persistence of both aggressor avoidance and reduced sociability in stressed mice. Thus, although the present variables (pecking behavior and feather damage) are distinct from Liu et al. (58), (sociability and avoidance), the effect of *L. rhamnosus* supplement could vary depending on whether it was administered before, during or after stress exposure. That said, the Lacto treatment did appear to be protective against stress-induced weight loss with no difference in body weight between stressed and non-stressed Lacto birds, while the stressed-placebo birds weighed 3% less than their non-stressed counterparts. Regardless, further work on the potential beneficial effects of *L. rhamnosus* supplementation, even under non-stressful conditions, should not be overlooked as intact feather cover has both biological and economic benefits, e.g., by improving bird welfare and reducing farm feed costs (2).

The proportion of T-cell subsets, except for the splenic T helper and Treg cells, were 2 to 3-fold lower than previously observed in younger birds (10 and 28 weeks) of similar lines (15, 41), which is most likely an age-related effect (59, 60). However, the dysregulation of T cell (without subset specification) proliferation and activation has been suggested to be the initial cause of the FP phenotype possibly via cholinergic signaling (61). Interestingly, we also report that tonsil Treg cells in severe feather peckers are 22% lower than in non-peckers (Supplementary Table 2). Tregs are a population of immunosuppressive CD4^+^ T lymphocytes involved in maintaining immune tolerance to self-antigens and preventing autoimmune/autoinflammatory disease (62). As such, Tregs help suppress inflammatory responses (63). Chicken Treg cells have suppressive properties similar to that of mammalian Treg cells (64) and depleted Treg populations have been associated with negative changes in mood and behavior in animal models (65–68). Our results support the idea that SFP could be an immune-related behavioral response, and more specifically, that Treg cells could play a role in determining SFP behavior.

We further report that *L. rhamnosus* supplementation had a strong immunomodulatory effect in laying hens, whereby it increased Treg cells and cytotoxic T cells in the spleen and cecal tonsils compared to birds receiving the Placebo. These results are consistent with previous mammalian (69–73) and avian studies (41, 42, 74). Apart from increased Treg cells, *L. rhamnosus* treatment also had anti-depressive and anxiolytic effects in mice (26, 73), and these effects were mediated through Treg cells (75). This implies a link between the immune response and behavior in mammals. To assess differences in reactivity behavior, we conducted a tonic immobility test, a well validated standardized test of fearfulness in chickens (76). We found that SFP and tonic immobility outcomes were correlated to the proportion of Treg cells in Lacto birds (data not shown), even if Lacto and stress treatment did not directly impact birds' fear responses. Indeed, severe feather peckers supplemented with Lacto had increased proportions of Treg cells in the tonsils and reduced level of SFP compared to the Placebo birds (data not shown). Additionally, low levels of splenic Tregs were associated with longer tonic immobility duration (r = −0.39, *P* = 0.034) and fewer induction of tonic immobility (r = 0.43, *P* = 0.019), suggesting a more fearful state. Similarly, a lower proportion of tonsil Tregs was associated with fewer tonic immobility inductions (r = 0.43, *P* = 0.017). These correlations were not significant in Placebo birds. Thus, we propose that *L. rhamnosus* oral supplementation positively modulates the immune system through Treg cell induction and that *L. rhamnosus* supplementation is positively linked to social interactions such as SFP behaviors and indirectly to fear in laying hens, mirroring work in mammalian models. Immunosuppressive Treg cells may then play an essential role in mediating the avian gut-brain axis signaling. It would be of further interest to investigate this hypothesis and test if the mechanism of T cell induction via *L. rhamnosus* supplementation is similar to mammals. In mice, *L. rhamnosus* is taken up by dendritic cells in the Peyer's patches, which then induce Treg cell production (71), a process that is vagus nerve-dependant (26).

Severe feather peckers had significantly lower peripheral TRP:(PHE+TYR) and higher TYR concentration than birds that did not express SFP (Supplementary Table 5). Previous studies unambiguously identified monoamine signaling as a key component in SFP behavior, mostly due to low central serotonin and dopamine turnover at a young age (12, 77, 78). We focused on AAA as these are the precursor metabolites for neurotransmitters of the serotonergic and catecholaminergic systems. We estimated their concentrations in the blood, and besides catabolism also transports affects the CNS availability, thus these both processes were taken into account by calculating relevant ratios. AAA concentrations were interesting to get first insights on the potential modulation of the crosstalk between the nutritional status and the serotonergic and catecholaminergic neurotransmitter biosynthesis upon *L. rhamnosus* supplementation and/or stress treatment. However, we found no variation of peripheral plasma concentrations of TRP, PHE, TYR, and KYN, their relevant ratios, and nitrite at 37 woa in response to 3 weeks of Lacto supplementation and stress. Previously, we demonstrated that early-age consumption of *L. rhamnosus* for 8 weeks led to a short-term increase of peripheral TRP concentrations and TRP:(PHE+TYR) in pullets (42). Amino acid and peptide absorption in birds (79) is similar to that in mammals (80). Still, during the first few weeks following hatch, the intestinal tract of birds grows rapidly (80). This may explain why no difference was observed in mature birds who were supplemented after the microbiome would be established in the current study (supplementation 33-38 woa) and previously reported (supplementation 19-28 woa) (41) compared to the study with pullets (supplemented 0-9 woa) (42). While *L. rhamnosus* has been shown to impact specific neurotransmitters-like GABA receptor expression (26), our results suggest that the effect on AAAs and related neurotransmitters pathways might be minimal, short-term or it suggests that the five weeks of *L. rhamnosus* supplementation may not be sufficiently long to impact monoamines precursors. However, it needs to be mentioned that there are still many unexplained variables, also regarding basic avian metabolism. For example, only IDO-2, which is less efficient in breaking down TRP along the KYN pathway than IDO-1, has been detected in birds and the regulation of the avian enzyme is still poorly understood in terms of modulation of activity by nutritional or potentially also stress or immunological factors (53).

It is noteworthy that the overall expression of SFP was scarce with an average frequency of 0.078 ± 0.555 pecks/bird/10 min across all birds between 35 and 38 woa. The observed level of SFP was approximately 7 fold lower than frequencies observed in 16-weeks old pullets (13) and 28–29 weeks old birds (11) of the same genetic HFP line. Despite pilot testing, it cannot be ruled out that the time window of behavior recording may not accurately reflect the true behavioral outcomes which may have impacted the results. Direct recording of behavior in large groups of birds is difficult and time consuming to perform, and scoring of feather cover provide a reliable estimation of the intensity of the behavior (54). Moreover, it can be argued that in commercial farms, feather damage, rather than the behavior itself, is of interest due to its aforementioned welfare and economic consequences. For this reason, we mainly focused on the feather damage outcome in the current study. However, because the purpose of our study was also to identify the physiological pathways linked to the behavior, we did record SFP during the experiment which allowed us to retrospectively determine bird phenotypes.

We additionally investigated the differences between the genetic lines. The present experiment mixed the HFP, LFP and UC genetic lines equally within the housing pens. This was done to mimic commercial conditions, but could have influenced the occurrence of feather damage and performance of SFP as LFP birds could be at higher risk of being victims or have learned to perform this behavior from the HFP birds (81) compared to if they were housed in groups according to their genetic line. This mixed housing of genetic lines could potentially also explain why the levels of feather damage were relatively high even in the non-stressed group. Additionally, this may partly explain the variability in the findings compared to Mindus et al. (41), who used only HFP birds. Indeed, differences in stress response (8, 10, 82), as well as in the reactivity of the immune system between various genetic lines (8, 15), are well documented. Similar to van der Eijk et al. (11), we found that HFP birds spent less time in and needed more inductions to be in tonic immobility, suggesting that they were less fearful than LFP birds (50, 76). Interestingly, LFP birds had more damaged feather cover than HFP birds, which may have enhanced their fearfulness. While we did not observe differences in fear response or feather damage to the Lacto or stress treatments between the genetic lines, we did observe that LFP birds stand out in their stress response in other physiological responses. Indeed, stress usually suppresses the avian immune system (55, 83, 84), an effect that was observed in the HFP and UC lines where, overall, all T cells proportions were lower in the stress groups (Table 2). On the contrary, the stress treatment increased the proportion of all T cells in the LFP line. Nevertheless, this increase was only significantly different for the splenic Treg cells (Table 2). Similarly, stress increased peripheral KYN concentrations and KYN:TRP in the LFP line, but not in the HFP and UC lines (Supplementary Table 4). These results may suggest that the LFP birds may be less sensitive to stress or that their stress-sensitive physiological pathways have a different regulation (85). These differences in stress response between the genetic lines were not, however, reflected in the SFP phenotype displayed from week 35 until blood/tissue collection at week 37–38. Thus, it is unclear whether they might play a role in SFP behavior. The causation of these differences should be further investigated to better understand the relationship between these physiological pathways and feather pecking behavior.

Mindus et al. (41) showed that individual Lacto supplementation of birds at 5 x 10^9^ CFU/bird prevented stress-induced SFP. In the present study, birds were treated with the *L. rhamnosus* supplement as a group to mimic commercial farm conditions which precludes individual administration of treatments as thousands of birds comprise a typical flock. We introduced the equivalent of 5 x 10^9^ CFU of *L. rhamnosus* per bird *via* two 1L-round drinkers and ensured that the full volume was consumed. This strategy prevented measuring individual consumption. Regardless of the individual dosage received, we observed strong and immediate immunomodulatory effects. Nevertheless, as *Lactobacillus* bacteria show some dose-dependent response in mammals (86–88), it is possible that this dosage was not sufficient to alter SFP behavior in the genetic lines of birds used in the present study. Similarly, individual feed consumption was not measured. Amino acids concentrations are largely controlled by total dietary intake, and thus, variations in feed consumption may have impacted the observed results.

## Conclusion

To study the impact of probiotic bacteria as an immediate measure against stress-induced feather damage, we supplemented adult laying hens with a daily dosage of *L. rhamnosus*, while following a validated stress regimen. We also investigated whether the immune and aromatic amino acids responses differed between the genetic lines and were interrelated with the severe feather-pecking phenotype itself to better understand the underlying physiological pathways of this behavior. Three weeks of stress treatment aggravated the severity of plumage damage. *L. rhamnosus* supplementation improved the birds' feather cover under non-stressful conditions; however, considering our previous study (41) *L. rhamnosus* supplementation needs to be provided ahead of stressful conditions. The severe feather-pecking phenotype was linked to lower proportions of regulatory T cells and lower TRP:(PHE+TYR). *L. rhamnosus* supplementation increased regulatory and cytotoxic T cells in the spleen and cecal tonsils, which were also correlated to birds' fear responses during tonic immobility. Thus, *L. rhamnosus* supplementation may modulate SFP and fearfulness via regulatory T cells induction. Our findings help elucidate biological mechanisms that are associated with SFP behavior and the pathways through which *L. rhamnosus* supplementation may mitigate behavior. These results pave the way for a better understanding of how individualized, microbial interventions can help reduce feather damage in commercial farms, and thus, improve the welfare of millions of domestic birds.

## Data Availability Statement

The raw data supporting the conclusions of this article will be made available by the authors, without undue reservation.

## Ethics Statement

The animal study was reviewed and approved by University of Guelph Animal Care Committee (Animal Utilization Protocol #4113).

## Author Contributions

CM, NS, WK, PF, and AH-M: conceived and designed the experiment. WK, PF, and AH-M: secured the funding for the experiment. CM and NS: performed the experiment and collected the data at the research station. DF, JG, and MM: processed the samples. CM: conducted the statistical analysis and wrote the original draft. CM, NS, DF, JG, JK, WK, MM, AS, PF, and AH-M: reviewed and approved the final manuscript before submission. All authors contributed to the article and approved the submitted version.

## Funding

This work was supported by the Natural Sciences and Engineering Research Council of Canada (NSERC)—Discovery Grant number 400983, the Ontario Ministry of Agriculture, Food and Rural Affairs (OMAFRA), Grant number 27267 and 2015-2384, and Egg Farmers of Canada. The funders had no role in the design of the study; in the collection, analyses, or interpretation of data; in the writing of the manuscript, and in the decision to publish the results.

## Conflict of Interest

The authors declare that the research was conducted in the absence of any commercial or financial relationships that could be construed as a potential conflict of interest.

## Publisher's Note

All claims expressed in this article are solely those of the authors and do not necessarily represent those of their affiliated organizations, or those of the publisher, the editors and the reviewers. Any product that may be evaluated in this article, or claim that may be made by its manufacturer, is not guaranteed or endorsed by the publisher.

## References

[1] Food Agriculture Organization of the United Nations. FAOSTAT Statistical Database. (2021) Available online at: https://www.fao.org/faostat/en/#data/QCL (accessed June 18, 2021).

[2] van Staaveren N Harlander A. Cause and prevention of injurious pecking in chickens. In: Nicol C, editor. Understanding the behaviour and improving the welfare of chickens. UK: Burleigh Dodds Series in Agricultural Science: Royal Veterinary College - University of London (2020).

[3] van Zeeland YRA Schoemaker NJ. Plumage disorders in psittacine birds - part 1: feather abnormalities. Eur J Companion Anim Pract. (2014) 24:34–47.

[4] LeBlanc C Tobalske B Bowley S Harlander-Matauschek A. Development of locomotion over inclined surfaces in laying hens. Animal. (2018) 12:585–96. 10.1017/S1751731117001896 28780926

[5] León BM Tobalske BW Sassi N. Ben, Garant R, Powers DR, Harlander-Matauschek A. Domestic egg-laying hens, Gallus gallus domesticus, do not modulate flapping flight performance in response to wing condition. R Soc Open Sci. (2021) 8:210196. 10.1098/rsos.210196 34350016 PMC8316787

[6] Bishop CM Butler BJ. Flight. In: Scanes CG, editor. Sturkie's Avian Physiology. 6th ed. London: Academic Press (2015). p. 919-74.

[7] Nicol C. 9. Feather Pecking and Cannibalism: Can We Really Stop Beak Trimming? In: Mench JA. UK: Woodhead Publishing. (2018). 10.1016/B978-0-08-100915-4.00009-9

[8] Brunberg EI Bas Rodenburg T Rydhmer L Kjaer JB Jensen P Keeling LJ. Omnivores going astray: A review and new synthesis of abnormal behavior in pigs and laying hens. Front Vet Sci. (2016) 3:1–15. 10.3389/fvets.2016.00057 27500137 PMC4956668

[9] Kjaer JB Sørensen P Su G. Divergent selection on feather pecking behaviour in laying hens (Gallus gallus domesticus). Appl Anim Behav Sci. (2001) 71:229–39. 10.1016/S0168-1591(00)00184-2 11230903

[10] van der Eijk JAJ Lammers A Kjaer JB Rodenburg TB. Stress response, peripheral serotonin and natural antibodies in feather pecking genotypes and phenotypes and their relation with coping style. Physiol Behav. (2019) 199:1–10. 10.1016/j.physbeh.2018.10.021 30391356

[11] van der Eijk JAJ Lammers A Li P Kjaer JB Rodenburg TB. Feather pecking genotype and phenotype affect behavioural responses of laying hens. Appl Anim Behav Sci. (2018) 205:141–50. 10.1016/j.applanim.2018.05.027

[12] de Haas EN van der Eijk JAJ. Where in the serotonergic system does it go wrong? Unravelling the route by which the serotonergic system affects feather pecking in chickens. Neurosci Biobehav Rev. (2018) 95:170–88. 10.1016/j.neubiorev.2018.07.007 30055196

[13] Birkl P Chow J Forsythe P Gostner JM Kjaer JB Kunze WA. The role of tryptophan-kynurenine in feather pecking in domestic chicken lines. Front Vet Sci. (2019) 6:209. 10.3389/fvets.2019.00209 31316999 PMC6610432

[14] Parmentier HK Rodenburg TB Reilingh GDV Beerda B Kemp B. Does enhancement of specific immune responses predispose laying hens for feather pecking? Poult Sci. (2009) 88:536–42. 10.3382/ps.2008-00424 19211522

[15] van der Eijk JAJ Verwoolde MB de Vries Reilingh G Jansen CA Rodenburg TB Lammers A. Chickens divergently selected on feather pecking differ in immune characteristics. Physiol Behav. (2019) 112680. 10.1016/j.physbeh.2019.112680 31518579

[16] Meyer B Zentek J Harlander-Matauschek A. Differences in intestinal microbial metabolites in laying hens with high and low levels of repetitive feather-pecking behavior. Physiol Behav. (2013) 110:96–101. 10.1016/j.physbeh.2012.12.017 23313560

[17] Meyer B Bessei AW Vahjen W Zentek J Harlander-Matauschek A. Dietary inclusion of feathers affects intestinal microbiota and microbial metabolites in growing Leghorn-type chickens. Poult Sci. (2012) 91:1506–13. 10.3382/ps.2011-01786 22700493

[18] Birkl P Bharwani A Kjaer JB Kunze W McBride P Forsythe P. Differences in cecal microbiome of selected high and low feather-pecking laying hens. Poult Sci. (2018) 97:3009–14. 10.3382/ps/pey167 29800328 PMC6093748

[19] van der Eijk JAJ de Vries H Kjaer JB Naguib M Kemp B Smidt H Rodenburg TB. Differences in gut microbiota composition of laying hen lines divergently selected on feather pecking. Poult Sci. (2019) 98:7009–21. 10.3382/ps/pez336 31226709 PMC6869756

[20] Apajalahti J Kettunen A Graham H. Characteristics of the gastrointestinal microbial communities, with special reference to the chicken. Worlds Poult Sci J. (2004) 60:223–32. 10.1079/WPS20040017

[21] Apajalahti J Kettunen A. Microbes of the chicken gastrointestinal tract. In: Perry GC. Avian Gut Function in Health and Disease. CAB International (2006). p. 124–137. 10.1079/9781845931803.0124

[22] Tannock GW. Minireviews a special fondness for lactobacilli. Appl Environ Microbiol. (2004) 70:3189–94. 10.1128/AEM.70.6.3189-3194.2004 15184111 PMC427720

[23] Ballou AL Ali RA Mendoza MA Ellis JC Hassan HM Croom WJ. Development of the chick microbiome: how early exposure influences future microbial diversity. Front Vet Sci. (2016) 3:2. 10.3389/fvets.2016.00002 26835461 PMC4718982

[24] Yong SJ Tong T Chew J Lim WL. Antidepressive mechanisms of probiotics and their therapeutic potential. Front Neurosci. (2020) 13. 10.3389/fnins.2019.01361 32009871 PMC6971226

[25] Forsythe P Sudo N Dinan T Taylor VH Bienenstock J. Mood and gut feelings. Brain Behav Immun. (2010) 24:9–16. 10.1016/j.bbi.2009.05.058 19481599

[26] Bravo JA Forsythe P Chew M V Escaravage E Savignac HM Dinan TG. Ingestion of Lactobacillus strain regulates emotional behavior and central GABA receptor expression in a mouse via the vagus nerve. Proc Natl Acad Sci. (2011) 108:16050–5. 10.1073/pnas.1102999108 21876150 PMC3179073

[27] McVey Neufeld K-AA O'Mahony SM Hoban AE Waworuntu R V Berg BM Dinan TG. Neurobehavioural effects of Lactobacillus rhamnosus GG alone and in combination with prebiotics polydextrose and galactooligosaccharide in male rats exposed to early-life stress. Nutr Neurosci. (2019) 22:425–34. 10.1080/1028415X.2017.1397875 29173065

[28] Palomar MM Maldonado Galdeano C Perdigón G. Influence of a probiotic lactobacillus strain on the intestinal ecosystem in a stress model mouse. Brain Behav Immun. (2014) 35:77–85. 10.1016/j.bbi.2013.08.015 24016865

[29] Huang R Wang K Hu J. Effect of probiotics on depression: A systematic review and meta-analysis of randomized controlled trials. Nutrients. (2016) 8:483. 10.3390/nu8080483 27509521 PMC4997396

[30] Lew LC Hor YY Yusoff NAA Choi SB Yusoff MSB Roslan NS. Probiotic Lactobacillus plantarum P8 alleviated stress and anxiety while enhancing memory and cognition in stressed adults: A randomised, double-blind, placebo-controlled study. Clin Nutr. (2019) 38:2053–64. 10.1016/j.clnu.2018.09.010 30266270

[31] Mindus C Ellis J van Staaveren N Harlander-Matauschek A. Lactobacillus-based probiotics reduce the adverse effects of stress in rodents: a meta-analysis. Front Behav Neurosci. (2021) 15:642757. 10.3389/fnbeh.2021.642757 34220459 PMC8241911

[32] Noujaim JC Andreatti Filho RL Lima ET Okamoto AS Amorim RL Torres Neto R. Detection of T lymphocytes in intestine of broiler chicks treated with Lactobacillus spp. and challenged with Salmonella enterica serovar enteritidis. Poult Sci. (2008) 87:927–33. 10.3382/ps.2007-00476 18420983

[33] Dec M Nowaczek A Urban-Chmiel R Stepień-Pyśniak D Wernicki A. Probiotic potential of Lactobacillus isolates of chicken origin with anti-Campylobacter activity. J Vet Med Sci. (2018) 80:1195–203. 10.1292/jvms.18-0092 29877314 PMC6115247

[34] Valladares R Bojilova L Potts AH Cameron E Gardner C Lorca G. Lactobacillus johnsonii inhibits indoleamine 2,3-dioxygenase and alters tryptophan metabolite levels in BioBreeding rats. FASEB J. (2013) 27:1711–20. 10.1096/fj.12-223339 23303207

[35] Marin IA Goertz JE Ren T Rich SS Onengut-Gumuscu S Farber E. Microbiota alteration is associated with the development of stress-induced despair behavior. Sci Rep. (2017) 7:1–10. 10.1038/srep43859 28266612 PMC5339726

[36] Gummalla S Broadbent JR. Tyrosine and phenylalanine catabolism by Lactobacillus cheese flavor adjuncts. J Dairy Sci. (2001) 84:1011–9. 10.3168/jds.S0022-0302(01)74560-2 11384026

[37] Fernstrom JD Fernstrom MH. Tyrosine, phenylalanine, and catecholamine synthesis and function in the brain. J Nutr. (2007) 137:1539S−1547S. 10.1093/jn/137.6.1539S 17513421

[38] Wurtman RJ Hefti F Melamed E. Precursor control of neurotransmitter synthesis. Pharmacol Rev. (1980) 32:315–35. 6115400

[39] Fuchs D Möller AA Reibnegger G Werner ER Werner-Felmayer G Dierich MP. Increased endogenous interferon-gamma and neopterin correlate with increased degradation of tryptophan in human immunodeficiency virus type 1 infection. Immunol Lett. (1991) 28:207–11. 10.1016/0165-2478(91)90005-U 1909303

[40] Neurauter G Schrocksnadel K Scholl-Burgi S Sperner-Unterweger B Schubert C Ledochowski M. Chronic immune stimulation correlates with reduced phenylalanine turnover. Curr Drug Metab. (2008) 9:622–7. 10.2174/138920008785821738 18781914

[41] Mindus C van Staaveren N Bharwani A Fuchs D Gostner JM Kjaer JB. Ingestion of Lactobacillus rhamnosus modulates chronic stress-induced feather pecking in chickens. Sci Rep. (2021) 11:17119. 10.1038/s41598-021-96615-x 34429482 PMC8384842

[42] Mindus C van Staaveren N Fuchs D Gostner JM Kjaer JB Kunze W. rhamnosus improves the immune response and tryptophan catabolism in laying hen pullets. Sci Rep. (2021) 11:19538. 10.1038/s41598-021-98459-x 34599202 PMC8486881

[43] Wu RY Pasyk M Wang B Forsythe P Bienenstock J Mao YK. Spatiotemporal maps reveal regional differences in the effects on gut motility for Lactobacillus reuteri and rhamnosus strains. Neurogastroenterol Motil. (2013) 25. 10.1111/nmo.12072 23316914

[44] van Staaveren N Krumma J Forsythe P Kjaer JB Kwon IY Mao Y-K. Cecal motility and the impact of Lactobacillus in feather pecking laying hens. Sci Rep. (2020) 10:12978. 10.1038/s41598-020-69928-6 32737381 PMC7395806

[45] West C Wu RY Wong A Stanisz AM Yan R Min KK. Lactobacillus rhamnosus strain JB-1 reverses restraint stress-induced gut dysmotility. Neurogastroenterol Motil. (2017) 29:1–11. 10.1111/nmo.12903 27381257

[46] Walker M Fureix C Palme R Newman JA Dallaire JA Mason G. Mixed-strain housing for female C57BL/6, DBA/2, and BALB/c mice: validating a split-plot design that promotes refinement and reduction. BMC Med Res Methodol. (2016) 16:11. 10.1186/s12874-016-0113-7 26817696 PMC4729181

[47] Huber-Eicher B Audige L. Analysis of risk factors for the occurrence of feather pecking in laying hen growers. Br Poult Sci. (1999) 40:599–604. 10.1080/00071669986963 10670670

[48] Harlander Matauschek A Beck P Rodenburg TB. Effect of an early bitter taste experience on subsequent feather-pecking behaviour in laying hens. Appl Anim Behav Sci. (2010) 127:108–14. 10.1016/j.applanim.2010.09.005

[49] Decina C Berke O van Staaveren N Baes CF Harlander-Matauscheck A. Development of a scoring system to assess feather damage in Canadian laying hen flocks. Animals. (2019) 9:436. 10.3390/ani9070436 31295882 PMC6680733

[50] Bryan Jones R Faure JM. Tonic immobility (“righting time”) in laying hens housed in cages and pens. Appl Anim Ethol. (1981) 7:369–72. 10.1016/0304-3762(81)90063-8

[51] Geisler S Mayersbach P Becker K Schennach H Fuchs D Gostner JM. Serum tryptophan, kynurenine, phenylalanine, tyrosine and neopterin concentrations in 100 healthy blood donors. Pteridines. (2015) 26:31–6. 10.1515/pterid-2014-0015

[52] Ball HJ Sanchez-Perez A Weiser S Austin CJD Astelbauer F Miu J. Characterization of an indoleamine 2,3-dioxygenase-like protein found in humans and mice. Gene. (2007) 396:203–13. 10.1016/j.gene.2007.04.010 17499941

[53] Yuasa HJ Mizuno K Ball HJ. Low efficiency IDO2 enzymes are conserved in lower vertebrates, whereas higher efficiency IDO1 enzymes are dispensable. FEBS J. (2015) 282:2735–45. 10.1111/febs.13316 25950090

[54] Bilčik B Keeling LJ. Changes in feather condition in relation to feather pecking and aggressive behaviour in laying hens. Br Poult Sci. (1999) 40:444–51. 10.1080/00071669987188 10579400

[55] Nazar FN Marin RH. Chronic stress and environmental enrichment as opposite factors affecting the immune response in Japanese quail (Coturnix coturnix japonica). Stress. (2011) 14:166–73. 10.3109/10253890.2010.523093 21034299

[56] McKean J Naug H Nikbakht E Amiet B Colson N. Probiotics and subclinical psychological symptoms in healthy participants: a systematic review and meta-analysis. J Altern Complement Med. (2017) 23:249–58. 10.1089/acm.2016.0023 27841940

[57] Messaoudi M Lalonde R Violle N Javelot H Desor D Nejdi A. Assessment of psychotropic-like properties of a probiotic formulation (*Lactobacillus helveticus* R0052 and *Bifidobacterium longum* R0175) in rats and human subjects. Br J Nutr. (2011) 105:755–64. 10.1017/S0007114510004319 20974015

[58] Liu Y Steinhausen K Bharwani A Mian MF Neufeld KAMV Forsythe P. Increased persistence of avoidance behaviour and social deficits with L rhamnosus JB-1 or selective serotonin reuptake inhibitor treatment following social defeat. Sci Rep. (2020) 10:1–13. 10.1038/s41598-020-75605-5 32778662 PMC7417579

[59] Kannan TA Ramesh G Ushakumari S Dhinakarraj G Vairamuthu S. Age related changes in T cell subsets in thymus and spleen of layer chicken (Gallus domesticus). Int J Curr Microbiol App Sci. (2017) 6:15–9. 10.20546/ijcmas.2017.601.002

[60] Bridle BW Julian R Shewen PE Vaillancourt JP Kaushik AK T. lymphocyte subpopulations diverge in commercially raised chickens. Can J Vet Res. (2006) 70:183–90. 16850940 PMC1477934

[61] Falker-Gieske C Mott A Preuß S Franzenburg S Bessei W Bennewitz J. Analysis of the brain transcriptome in lines of laying hens divergently selected for feather pecking. BMC Genomics. (2020) 21:1–14. 10.1186/s12864-020-07002-1 32854615 PMC7457272

[62] Corthay A. How do regulatory T cells work? Scand J Immunol. (2009) 70:306–36. 10.1111/j.1365-3083.2009.02308.x 19751267 PMC2784904

[63] Sakaguchi S Yamaguchi T Nomura T Ono M. Regulatory T cells and immune tolerance. Cell. (2008) 133:775–87. 10.1016/j.cell.2008.05.009 18510923

[64] Shanmugasundaram R Selvaraj RK. Regulatory T Cell Properties of Chicken CD4 + CD25 + Cells. J Immunol. (2011) 186:1997–2002. 10.4049/jimmunol.1002040 21242520

[65] Ellul P Mariotti-Ferrandiz E Leboyer M Klatzmann D. Regulatory T cells as supporters of psychoimmune resilience: toward immunotherapy of major depressive disorder. Front Neurol. (2018) 9:1. 10.3389/fneur.2018.00167 29615964 PMC5869201

[66] Sommershof A Aichinger H Engler H Adenauer H Catani C Boneberg EM. Substantial reduction of naïve and regulatory T cells following traumatic stress. Brain Behav Immun. (2009) 23:1117–24. 10.1016/j.bbi.2009.07.003 19619638

[67] Kim S-J Lee H Lee G Oh S-J Shin M-K Shim I. CD4+CD25+ regulatory T cell depletion modulates anxiety and depression-like behaviors in mice. PLoS ONE. (2012) 7:e42054. 10.1371/journal.pone.0042054 22860054 PMC3409145

[68] Cohen H Ziv Y Cardon M Kaplan Z Matar MA Gidron Y. Maladaptation to mental stress mitigated by the adaptive immune system via depletion of naturally occurring regulatory CD4+CD25+ cells. J Neurobiol. (2006) 66:552–63. 10.1002/neu.20249 16555237

[69] Jang S-O Kim H-J Kim Y-J Kang M-J Kwon J-W Seo J-H. Asthma prevention by lactobacillus rhamnosus in a mouse model is associated with CD4+CD25+ Foxp3+ T Cells. Allergy Asthma Immunol Res. (2012) 4:150–6. 10.4168/aair.2012.4.3.150 22548208 PMC3328732

[70] Karimi K Inman MD Bienenstock J Forsythe P. Lactobacillus reuteri-induced regulatory T cells protect against an allergic airway response in mice. Am J Respir Crit Care Med. (2009) 179:186–93. 10.1164/rccm.200806-951OC 19029003

[71] Karimi K Kandiah N Chau J Bienenstock J Forsythe PA. Lactobacillus rhamnosus Strain Induces a Heme Oxygenase Dependent Increase in Foxp3+ Regulatory T Cells. PLoS ONE. (2012) 7:1–12. 10.1371/journal.pone.0047556 23077634 PMC3471882

[72] Feleszko W Jaworska J Rha RD Steinhausen S Avagyan A Jaudszus A. Probiotic-induced suppression of allergic sensitization and airway inflammation is associated with an increase of T regulatory-dependent mechanisms in a murine model of asthma. Clin Exp Allergy. (2007) 37:498–505. 10.1111/j.1365-2222.2006.02629.x 17430345

[73] Bharwani A Mian MF Surette MG Bienenstock J Forsythe P. Oral treatment with Lactobacillus rhamnosus attenuates behavioural deficits and immune changes in chronic social stress. BMC Med. (2017) 15:1–14. 10.1186/s12916-016-0771-7 28073366 PMC5225647

[74] Khan S Moore RJ Stanley D Chousalkar KK. The gut microbiota of laying hens and its manipulation with prebiotics and probiotics to enhance gut health and food safety. Appl Environ Microbiol. (2020) 86. 10.1128/AEM.00600-20 32332137 PMC7301851

[75] Liu Y Mian MF McVey Neufeld KA Forsythe P. CD4+CD25+ T Cells are Essential for Behavioral Effects of Lactobacillus rhamnosus JB-1 in Male BALB/c mice. Brain Behav Immun. (2020) 88:451–60. 10.1016/j.bbi.2020.04.014 32276029

[76] Forkman B Boissy A Meunier-Salaün M-C Canali E Jones RB A. critical review of fear tests used on cattle, pigs, sheep, poultry and horses. Physiol Behav. (2007) 92:340–74. 10.1016/j.physbeh.2007.03.016 18046784

[77] Kops MS Kjaer JB Güntürkün O Westphal KGC Korte-Bouws GAH Olivier B. Brain monoamine levels and behaviour of young and adult chickens genetically selected on feather pecking. Behav Brain Res. (2017) 327:11–20. 10.1016/j.bbr.2017.03.024 28347825

[78] Kops MS de Haas EN Rodenburg TB Ellen ED Korte-Bouws GAH Olivier B. Effects of feather pecking phenotype (severe feather peckers, victims and non-peckers) on serotonergic and dopaminergic activity in four brain areas of laying hens (Gallus gallus domesticus). Physiol Behav. (2013) 120:77–82. 10.1016/j.physbeh.2013.07.007 23911692

[79] Gilbert ER Wong EA Webb KEJ. Board-invited review: Peptide absorption and utilization: Implications for animal nutrition and health. J Anim Sci. (2008) 86:2135–55. 10.2527/jas.2007-0826 18441086

[80] Denbow DM. Gastrointestinal Anatomy and Physiology. In: Scanes GC, editor. Sturkie's Avian Physiology: Sixth Edition. San Diego, USA. (2015) p. 337–366. 10.1016/B978-0-12-407160-5.00014-2

[81] Zeltner E Klein T Huber-Eicher B. Is there social transmission of feather pecking in groups of laying hen chicks? Anim Behav. (2000) 60:211–6. 10.1006/anbe.2000.1453 10973723

[82] Kjaer JB Guémené D. Adrenal reactivity in lines of domestic fowl selected on feather pecking behavior. Physiol Behav. (2009) 96:370–3. 10.1016/j.physbeh.2008.10.023 19027766

[83] Shini S Huff GR Shini A Kaiser P. Understanding stress-induced immunosuppression: Exploration of cytokine and chemokine gene profiles in chicken peripheral leukocytes. Poult Sci. (2010) 89:841–51. 10.3382/ps.2009-00483 20308420

[84] Trout JM Mashaly MM. Effects of in vitro corticosterone on chicken T and B-lymphocyte proliferation. Br Poult Sci. (1995) 36:813–20. 10.1080/00071669508417826 8746983

[85] Kjaer JB Jørgensen H. Heart rate variability in domestic chicken lines genetically selected on feather pecking behavior. Genes, Brain Behav. (2011) 10:747–55. 10.1111/j.1601-183X.2011.00713.x 21682845

[86] Gill HS Rutherfurd KJ. Viability and dose–response studies on the effects of the immunoenhancing lactic acid bacterium Lactobacillus rhamnosus in mice. Br J Nutr. (2001) 86:285–9. 10.1079/BJN2001402 11502243

[87] Zhu YH Li XQ Zhang W Zhou D Liu HY Wang JF. Dose-dependent effects of Lactobacillus rhamnosus on serum interleukin-17 production and intestinal T-cell responses in pigs challenged with Escherichia coli. Appl Environ Microbiol. (2014) 80:1787–98. 10.1128/AEM.03668-13 24389928 PMC3957626

[88] Evrard B Coudeyras S Dosgilbert A Charbonnel N Alamé J Tridon A. Dose-dependent immunomodulation of human dendritic cells by the probiotic lactobacillus rhamnosus lcr35. PLoS ONE. (2011) 6:1–12. 10.1371/journal.pone.0018735 21533162 PMC3078917

