# Supplementary material for: Regulatory T Cell Modulation by Lactobacillus rhamnosus Improves Feather Damage in Chickens
Source: Front Vet Sci. 2022 Apr 11;9:855261. doi: 10.3389/fvets.2022.855261 (PMC9036099; doi:10.3389/fvets.2022.855261)
Supplement: Supplementary file 1 [file Data_Sheet_1.docx]

Supplementary Material

**Supplementary Table 1**. Least squares means (± standard error) of the duration of immobility and means (± standard deviation) of the number of inductions in the tonic immobility test. Tonic immobility was conducted at 36 weeks of age following supplementation (*L. rhamnosus* [Lacto] or water [Placebo] supplementation, week 33-38) and stress treatment (stress or non-stress, week 33-35). No statistically significant difference was found due to the stress treatment, L. *rhamnosus* supplementation or their interaction. F-statistics and P-value of the supplementation x stress interaction are indicated.

|  | **Lacto** | | **Placebo** | | |  | |  | |  |
| --- | --- | --- | --- | --- | --- | --- | --- | --- | --- | --- |
|  | **Stress**  (n=79) | **Non-stress**  (n=78) | | **Stress**  (n=77) | **Non-stress**  (n=77) | | **F-Statistic** | | **P-value** | |
| Duration (sec) | 76.0 ± 13.1 | 72.1 ± 12.0 | | 63.4 ± 10.5 | 79.4 ± 13.2 | | F_1, 283_= 1.36 | | P=0.245 | |
| No. of inductions | 1.6 ± 0.91 | 1.7 ± 1.00 | | 1.5 ± 0.84 | 1.5 ± 0.84 | | F_1, 288_= 0.21 | | P=0.646 | |

**Supplementary Table 2.** Proportions of T cell sub-populations in the cecal tonsils and spleen of 38-weeks old laying hens according to their genetic line (UC: unselected control, LFP: low feather pecking line, HFP: high feather pecking line) and severe feather pecking phenotype (bird that displayed severe feather pecking at least once between 35-38 weeks of age regardless of genotype). Sub-populations were identified using the following combinations of cell surface markers: T helper cells = CD3^+^CD4^+^; cytotoxic T cells = CD3^+^CD8^+^; regulatory T cells = CD4^+^CD25^+^. Different superscript letters within each row indicate statistically significant differences (P<0.05).

|  | **UC** (n=20) | **LFP** (n=20) | **HFP** (n=20) | **F-Statistic, P-value** |
| --- | --- | --- | --- | --- |
| **Cecal Tonsils** |  |  |  |  |
| T helper cell | 13.9 ± 0.61 | 14.1 ± 0.62 | 14.0 ± 0.62 | F_2,47_=0.03, P=0.970 |
| Cytotoxic T cell | 13.7 ± 0.50 | 13.5 ± 0.50 | 13.2 ± 0.50 | F_2,47_=0.27, P=0.763 |
| Regulatory T cell | 16.3 ± 0.76 | 16.2 ± 0.76 | 14.5 ± 0.76 | F_2,47_=1.81, P=0.175 |
| **Spleen** |  |  |  |  |
| T helper cell | 15.6 ± 1.00 | 16.1 ± 1.11 | 14.4 ± 0.99 | F_2,47_=1.73, P=0.188 |
| Cytotoxic T cell | 20.8 ± 1.47 | 21.3 ± 1.53 | 19.9 ± 1.53 | F_2,47_=0.35, P=0.709 |
| Regulatory T cell | 20.5 ± 0.84 ^a^ | 17.4 ± 0.84 ^b^ | 17.6 ± 0.84 ^b^ | **F_2,47_=4.24, P=0.020** |
|  |  |  |  |  |
|  |  | **Severe Feather Pecker** (n=11) | **Non-Severe Feather Pecker** (n=45) | **F-Statistic, P-value** |
| **Cecal Tonsils** |  |  |  |  |
| T helper cell |  | 13.5 ± 0.82 | 13.9 ± 0.41 | F_1,53_=0.16, P=0.695 |
| Cytotoxic T cell |  | 13.6 ± 1.11 | 13.9 ± 0.69 | F_1,53_=0.08, P=0.773 |
| Regulatory T cell |  | 12.9 ± 1.58 ^a^ | 16.5 ± 0.78 ^b^ | **F_1,53_=4.06, P=0.049** |
| **Spleen** |  |  |  |  |
| T helper cell |  | 12.9 ± 0.94 ^a^ | 14.9 ± 0.54 ^b^ | **F_1,53_=4.07, P=0.049** |
| Cytotoxic T cell |  | 19.5 ± 2.05 | 20.3 ± 1.09 | F_1,53_=0.13, P=0.719 |
| Regulatory T cell |  | 18.4 ± 1.88 | 18.9 ± 0.93 | F_1,53_=0.05, P=0.831 |

**Supplementary Table 3.** Least squares means (± standard error) of concentrations of amino acids tryptophan (TRP), tyrosine (TYR) and phenylalanine (PHE), the TRP metabolite (kynurenine [KYN]), and nitrite in 37-week old laying hens. The birds underwent 5 weeks of supplementation (Lacto: *L. rhamnosus* supplementation*,* Placebo: placebo supplementation, 33-38 weeks of age) and 3 weeks of stress treatment (stress or non-stress, 33-35 weeks of age). No statistically significant difference was found due to the *L.* *rhamnosus* supplementation, stress treatment or their interaction. F-statistics and P-values of the supplementation x stress interaction are indicated.

|  | **Lacto** | | **Placebo** | |  |
| --- | --- | --- | --- | --- | --- |
|  | **Stress**  (n=79) | **Non-Stress**  (n=78) | **Stress**  (n=77) | **Non-Stress**  (n=77) | **F statistic, P-value** |
| Tryptophan (µmol/L) | 86 ± 1.9 | 85 ± 1.9 | 85 ± 1.9 | 87 ± 1.9 | F_1,277_=1.55, P=0.214 |
| Kynurenine (µmol/L) | 0.33 ± 0.019 | 0.33 ± 0.019 | 0.32 ± 0.019 | 0.28 ± 0.019 | F_1,277_=1.73, P=0.189 |
| Tyrosine (µmol/L) | 123 ± 4.0 | 119 ± 3.9 | 119 ± 4.0 | 121 ± 4.0 | F_1,274_=0.53, P=0.468 |
| Phenylalanine (µmol/L) | 108 ± 2.5 | 106 ± 2.5 | 105 ± 2.6 | 107 ± 2.5 | F_1,274_=0.51, P=0.477 |
| TRP:(PHE+TYR) (µmol/µmol) | 0.377 ± 0.0088 | 0.378 ± 0.0088 | 0.381 ± 0.0089 | 0.382 ± 0.0089 | F_1,272_=0.00, P=0.992 |
| KYN:TRP (µmol/mmol) | 3.8 ± 0.23 | 3.9 ± 0.23 | 3.8 ± 0.23 | 3.2 ± 0.23 | F_1,277_=2.20, P=0.139 |
| PHE:TYR (µmol/µmol) | 0.87 ± 0.014 | 0.88 ± 0.014 | 0.89 ± 0.015 | 0.89 ± 0.015 | F_1,274_=0.20, P=0.655 |
| Nitrite (µmol/L) | 72 ± 3.1 | 71 ± 3.1 | 69 ± 3.1 | 68 ± 3.2 | F_1,268_=0.00, P=0.990 |

**Supplementary Table 4.** Least squares means (± standard error) of amino acids tryptophan (TRP), tyrosine (TYR) and phenylalanine (PHE), the TRP metabolite (kynurenine [KYN]), and nitrite in 37-week old laying hens. The birds underwent 5 weeks of supplementation (33-38 weeks of age, Lacto: *L. rhamnosus* supplementation*,* Placebo: placebo supplementation) and 3 weeks of stress treatment (stress or non-stress, 33-35 weeks of age). Different superscript letters indicate statistically significant different comparisons within the interaction in each row. F-Statistics and P-value of the supplementation x line or stress x line interaction are indicated.

|  | **Lacto** | | | **Placebo** | | |  |
| --- | --- | --- | --- | --- | --- | --- | --- |
|  | **UC**  (n=60) | **LFP** (n=59) | **HFP** (n=58) | **UC**  (n=59) | **LFP** (n=59) | **HFP** (n=57) | **F-Statistic, P-value** |
| Tryptophan (µmol/L) | 81.7 ± 1.8 ^b^ | 88.5 ± 1.8 ^a^ | 85.6 ± 1.8 ^ab^ | 86.4 ± 1.8 ^ab^ | 86.3 ± 1.8 ^ab^ | 85.4 ± 1.8 ^ab^ | F_2,277_=3.02, P=0.050 |
| Kynurenine (µmol/L) | 0.33 ± 0.021 | 0.35 ± 0.021 | 0.32 ± 0.021 | 0.28 ± 0.020 | 0.33 ± 0.022 | 0.30 ± 0.021 | F_2,277_=0.33, P=0.719 |
| Tyrosine (µmol/L) | 122 ± 3.9 | 121 ± 4.0 | 121 ± 3.9 | 118 ± 3.8 | 125 ± 4.3 | 116 ± 3.9 | F_2,274_=0.93, P=0.396 |
| Phenylalanine (µmol/L) | 106 ± 2.6 | 107 ± 2.6 | 108 ± 2.6 | 106 ± 2.6 | 107 ± 2.8 | 105 ± 2.7 | F_2,274_=0.24, P=0.786 |
| TRP:(PHE+TYR)(µmol/µmol) | 0.361 ± 0.0089 | 0.392 ± 0.0091 | 0.379 ± 0.0090 | 0.387 ± 0.0088 | 0.374 ± 0.0097 | 0.384 ± 0.0093 | **F_2,272_=3.64, P=0.003** |
| KYN:TRP (µmol/mmol) | 4.0 ± 0.24 | 3.9 ± 0.25 | 3.6 ± 0.24 | 3.2 ± 0.24 | 3.8 ± 0.25 | 3.4 ± 0.25 | F_2,277_=1.27, P=0.282 |
| PHE:TYR (µmol/µmol) | 0.87 ± 0.017 | 0.89 ± 0.018 | 0.88 ± 0.017 | 0.89 ± 0.017 | 0.86 ± 0.019 | 0.9 ± 0.018 | F_2,274_=0.92, P=0.399 |
| Nitrite (µmol/L) | 68 ± 3.8 | 76 ± 3.8 | 69 ± 3.8 | 67 ± 3.6 | 64 ± 4.0 | 73 ± 3.9 | F_2,268_=2.21, P=0.112 |
|  |  |  |  |  |  |  |  |
|  | **Stress** | | | **Non-Stress** | | |  |
|  | **UC**  (n=59) | **LFP** (n=60) | **HFP** (n=58) | **UC**  (n=60) | **LFP** (n=58) | **HFP** (n=57) | **F-Statistic, P-value** |
| Tryptophan (µmol/L) | 83 ± 1.8 | 88 ± 1.8 | 86 ± 1.8 | 85 ± 1.8 | 87 ± 1.8 | 85 ± 1.8 | F_2,277_=0.88, P=0.417 |
| Kynurenine (µmol/L) | 0.29 ± 0.020 ^b^ | 0.38 ± 0.022 ^a^ | 0.31 ± 0.021 ^ab^ | 0.32 ± 0.021 ^ab^ | 0.29 ± 0.021 ^b^ | 0.30 ± 0.021 ^ab^ | **F_2,277_=4.69, P<0.01** |
| Tyrosine (µmol/L) | 120 ± 3.8 | 124 ± 4.3 | 118 ± 3.9 | 120 ± 3.9 | 121 ± 4.0 | 119 ± 3.9 | F_2,274_=0.21, P=0.815 |
| Phenylalanine (µmol/L) | 107 ± 2.6 | 108 ± 2.7 | 105 ± 2.6 | 105 ± 2.6 | 106 ± 2.7 | 107 ± 2.6 | F_2,274_=0.40, P=0.673 |
| TRP:(PHE+TYR)(µmol/µmol) | 0.369 ± 0.0088 | 0.383 ± 0.0095 | 0.385 ± 0.0091 | 0.379 ± 0.0089 | 0.384 ± 0.0093 | 0.377 ± 0.0092 | F_2,272_=0.66, P=0.516 |
| KYN:TRP (µmol/mmol) | 3.5 ± 0.24 ^ab^ | 4.3 ± 0.25 ^a^ | 3.5 ± 0.25 ^ab^ | 3.7 ± 0.24 ^ab^ | 3.3 ± 0.25 ^b^ | 3.5 ± 0.25 ^ab^ | **F_2,277_=4.68, P=0.01** |
| PHE:TYR (µmol/µmol) | 0.89 ± 0.017 | 0.87 ± 0.018 | 0.88 ± 0.018 | 0.88 ± 0.017 | 0.88 ± 0.018 | 0.9 ± 0.018 | F_2,274_=0.22, P=0.800 |
| Nitrite (µmol/L) | 68 ± 3.7 | 67 ± 4.0 | 76 ± 3.8 | 68 ± 3.8 | 73 ± 3.9 | 66 ± 3.9 | F_2,268_=2.00, P=0.137 |

**Supplementary Table 5.** Least squares means (± standard error) of amino acids tryptophan (TRP), tyrosine (TYR) and phenylalanine (PHE), the TRP metabolite (kynurenine [KYN]), and nitrite in 37-weeks old laying hens according to their genetic line (UC: unselected control, LFP: low feather pecking line, HFP: high feather pecking line) and severe feather pecking phenotype (bird that displayed severe FP at least once between 35-37 weeks of age regardless of genotype). Different superscript letters within each row indicate statistically significant differences (P<0.05).

|  | **UC** (n=106) | **LFP** (n=105) | **HFP** (n=100) | **F-Statistic, P-value** |
| --- | --- | --- | --- | --- |
| Tryptophan (µmol/L) | 84 ± 1.3 | 87 ± 1.3 | 85 ± 1.3 | F_2,277_=2.58, P=0.078 |
| Kynurenine (µmol/L) | 0.30 ± 0.014 | 0.34 ± 0.015 | 0.31 ± 0.015 | F_2,277_=1.90, P=0.152 |
| Tyrosine (µmol/L) | 120 ± 2.7 | 123 ± 2.9 | 119 ± 2.8 | F_2,274_=0.69, P=0.502 |
| Phenylalanine (µmol/L) | 106 ± 1.8 | 107 ± 1.9 | 106 ± 1.9 | F_2,274_=0.10, P=0.902 |
| TRP:(PHE+TYR) (µmol/µmol) | 0.37 ± 0.006 | 0.38 ± 0.007 | 0.38 ± 0.007 | F_2,272_=0.72, P=0.489 |
| KYN:TRP (µmol/mmol) | 3.6 ± 0.17 | 3.8± 0.18 | 3.5 ± 0.17 | F_2,277_=0.95, P=0.390 |
| PHE:TYR (µmol/µmol) | 0.88 ± 0.012 | 0.87 ± 0.013 | 0.89 ± 0.012 | F_2,274_=0.29, P=0.745 |
| Nitrite (µmol/L) | 68 ± 2.6 | 70 ± 2.8 | 71 ± 2.7 | F_2,268_=0.35, P=0.702 |
|  |  |  |  |  |
|  |  | **Severe Feather Pecker** (n=57) | **Non-Severe Feather Pecker** (n=239) | **F-Statistic, P-value** |
| Tryptophan (µmol/L) |  | 83.7 ± 1.43 | 86.2 ± 0.70 | F_1,293_= 2.39, P=0.123 |
| Kynurenine (µmol/L) |  | 0.287 ± 0.0188 | 0.320 ± 0.0095 | F_1,292_=2.42, P=0.121 |
| Tyrosine (µmol/L) |  | 128 ± 3.5 | 122 ± 2.2 | **F_1,288_=3.72, P=0.055** |
| Phenylalanine (µmol/L) |  | 110 ± 2.2 | 106 ± 1.1 | F_1,292_= 2.66, P=0.104 |
| TRP:(PHE+TYR) (µmol/µmol) |  | 0.35 ± 0.010 ^a^ | 0.38 ± 0.007 ^b^ | **F_1,292_=9.70, P=0.002** |
| KYN:TRP (µmol/mmol) |  | 3.4 ± 0.23 | 3.6 ± 0.14 | F_1,293_= 1.22, P=0.271 |
| PHE:TYR (µmol/µmol) |  | 0.871 ± 0.0172 | 0.882 ± 0.0083 | F_1,291_= 0.37, P=0.542 |
| Nitrite (µmol/L) |  | 71 ± 3.7 | 70 ± 2.1 | F_1,287_= 0.05, P=0.823 |
